# Common and Uncommon Splenic Lesions: A Review

**DOI:** 10.3390/diagnostics16152462

**Published:** 2026-08-04

**Authors:** Rajvir Teja, Evan Allarie, Christopher Fung

**Affiliations:** 1 Faculty of Medicine and Dentistry, University of Alberta, Edmonton, AB T6G 2B7, Canada; teja@ualberta.ca; 2 Department of Radiology and Diagnostic Imaging, University of Alberta, Edmonton, AB T6G 2B7, Canada; eallarie@ualberta.ca

**Keywords:** spleen, incidental finding, cyst, MRI, CT, ultrasound

## Abstract

**Background/Objectives**: Briefly review the imaging and pathologic findings associated with common and uncommon splenic lesions. These lesions are often incidental and cause a diagnostic dilemma in determining the need for further work-up and follow-up. **Methods/Results**: A step-by-step review of splenic lesions will be provided. This includes general demographics, fundamental pathology, and key imaging features. **Conclusions**: Splenic lesions are often challenging due to their relatively uncommon presentation, predominantly incidental detection, and broad age of incidence. The vast majority of these lesions, however, are benign. Correlation with the patient demographics and specific elements of the patient history will often significantly narrow the differential diagnosis.

## 1. Introduction

Incidentally detected splenic lesions are common and can pose a diagnostic conundrum. A structured approach to imaging interpretation—including evaluating patient demographics, history, and symptomatology—can guide accurate diagnoses, avoiding unnecessary imaging and invasive work-up. Physicians should be aware that the vast majority of incidental splenic lesions are benign in etiology [1]. However, overlapping imaging characteristics can make the definitive diagnosis of a given splenic lesion challenging. The 2026 Canadian guidelines help provide a framework for multimodality assessment of incidental splenic lesions [2]. These guidelines state that even an indeterminate lesion at the time of CT or MRI, in the absence of known malignancy/lymphoproliferative disease, and absence of constitutional symptoms, does not require dedicated follow-up imaging. The purpose of this review is to discuss key imaging features of incidental splenic lesions, with the goal of accurately recognizing actionable findings while avoiding unnecessary investigations and interventions.

## 2. Review of Splenic Lesions

### 2.1. Primary (Epithelial) Cysts

Primary cysts are also known as epithelial or true cysts. Primary cysts of the spleen account for 10% of all benign nonparasitic splenic cysts. The cysts form from mesothelial invagination during development [3]. The incidence of these benign lesions is increasing with the increasing role of imaging in clinical care [4]. These cysts are usually asymptomatic and discovered incidentally. They are most frequently found in children and young adults, with a slight predominance in females [5]. On pathology, these cysts have a smooth cystic wall composed of fibrous tissue that contains an epithelial lining of stratified, squamous, columnar, or cuboidal epithelium. The cysts are often filled with internal serous or proteinaceous fluid. The key pathologic feature is the presence of an epithelial lining, distinguishing primary (true cysts) from secondary cysts, which lack a lining [4].

**Figure 1:**
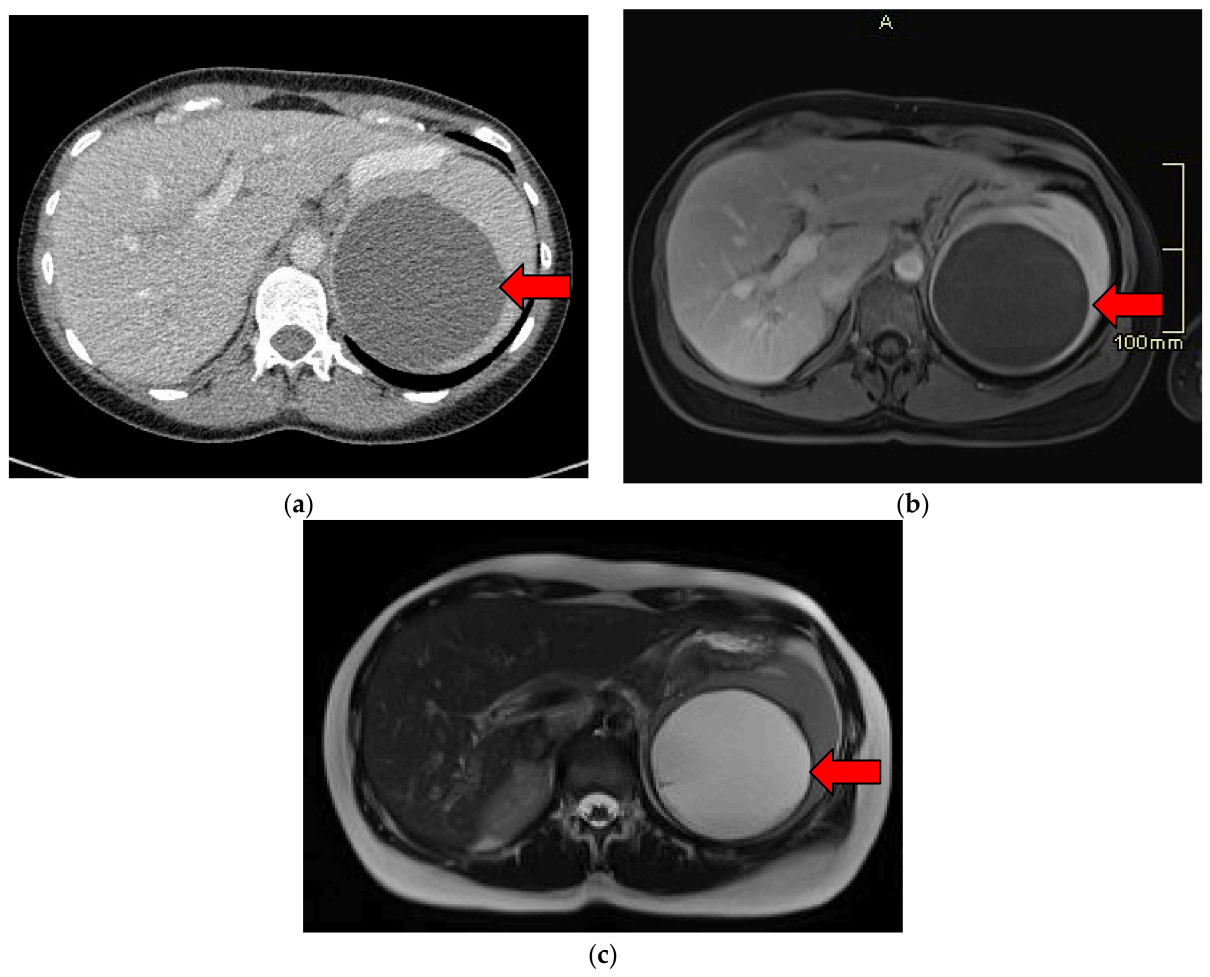
10 cm epithelial cyst in a 35-year-old female. (**a**) On this axial contrast-enhanced CT, the cyst is a homogenous thin-walled mass that is well circumscribed. (**b**) On this axial T1-weighted image, the cyst is a thin-walled, well-circumscribed mass that has homogeneously low signal intensity. (**c**) On this axial T2-weighted image, the cyst is a thin-walled, well-circumscribed mass with a homogeneously high signal.

Primary splenic cysts are well-defined, unilocular or multilocular, thin-walled anechoic lesions on ultrasound with no internal solid components or vascularity [6]. The lesions correspondingly do not show enhancement with contrast-enhanced ultrasound [7]. Primary splenic cysts rarely calcify [4]. On CT, they are characterized by homogenous low attenuation thin-walled masses with sharply demarcated borders (Figure 1a). Red arrows in all images indicate location of lesions. MRI demonstrates homogenous, high signal intensity on T2-weighted images and low signal intensity on T1-weighted images as seen in Figure 1b,c [6].

### 2.2. Pseudocysts

Splenic pseudocysts account for 70–80% of all nonparasitic splenic cysts and are more common than true cysts [4]. These lesions affect women more than men [8]. A defining pathological feature of pseudocysts is the absence of epithelial cells along the cyst lining, which is instead composed of dense fibrous tissue. These lesions are usually due to previous splenic trauma or infection [9].

**Figure 2:**
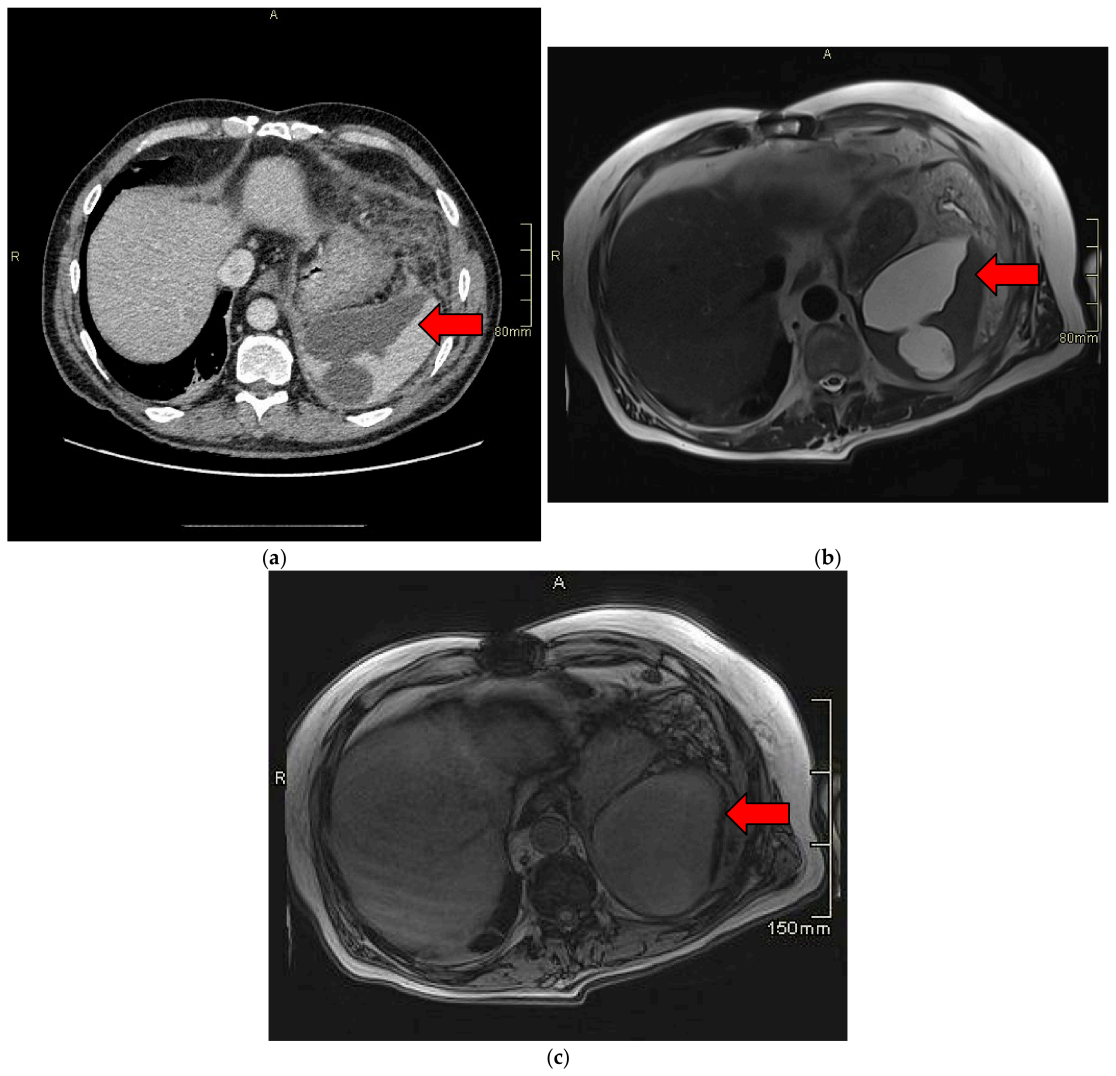
Pseudocyst in a 65-year-old male as a complication of acute pancreatitis (**a**) Axial contrast-enhanced CT of a well-circumscribed and unilocular hypodense pseudocyst with no overt calcifications or solid components. (**b**,**c**) Axial MRI T2-weighted image demonstrates a well-circumscribed, thin-walled, hyperintense splenic pseudocyst with no internal solid components.

On imaging, pseudocysts are well defined and hypo-echoic on ultrasound. There may be internal echogenic debris with hemorrhage and necrosis. Contrast-enhanced ultrasound shows no internal enhancement [10]. On CT, pseudocysts can be thick-walled and calcified, differentiating them from congenital cysts [4]. These lesions are generally well circumscribed. They may be unilocular or multilocular with fluid attenuation (Figure 2a). Pseudocysts on MRI can present with variable T1 intensity, depending on the contents being proteinaceous or hemorrhagic, and are hyperintense on T2-weighted images with no contrast enhancement in the wall or contents as seen in Figure 2b,c [4].

### 2.3. Hydatid Cyst

Splenic hydatid cysts account for 0.5–0.8% of all echinococcal presentations [11]. These parasitic lesions are more common outside of North America, in areas such as South America, the Mediterranean, the Middle East, Africa, and Australia [4]. The spleen is the third most commonly involved organ after the liver and lungs. Splenic hydatid cysts can occur at any age; they are most commonly reported in the fourth to fifth decades of life, with cases described in adults and children [12]. Splenic hydatid cysts are characterized pathologically by well-defined, unilocular or multilocular cystic lesions with a laminated acellular outer wall and an inner germinal layer, often containing clear fluid and daughter cysts. The cyst wall may show calcification [13].

**Figure 3:**
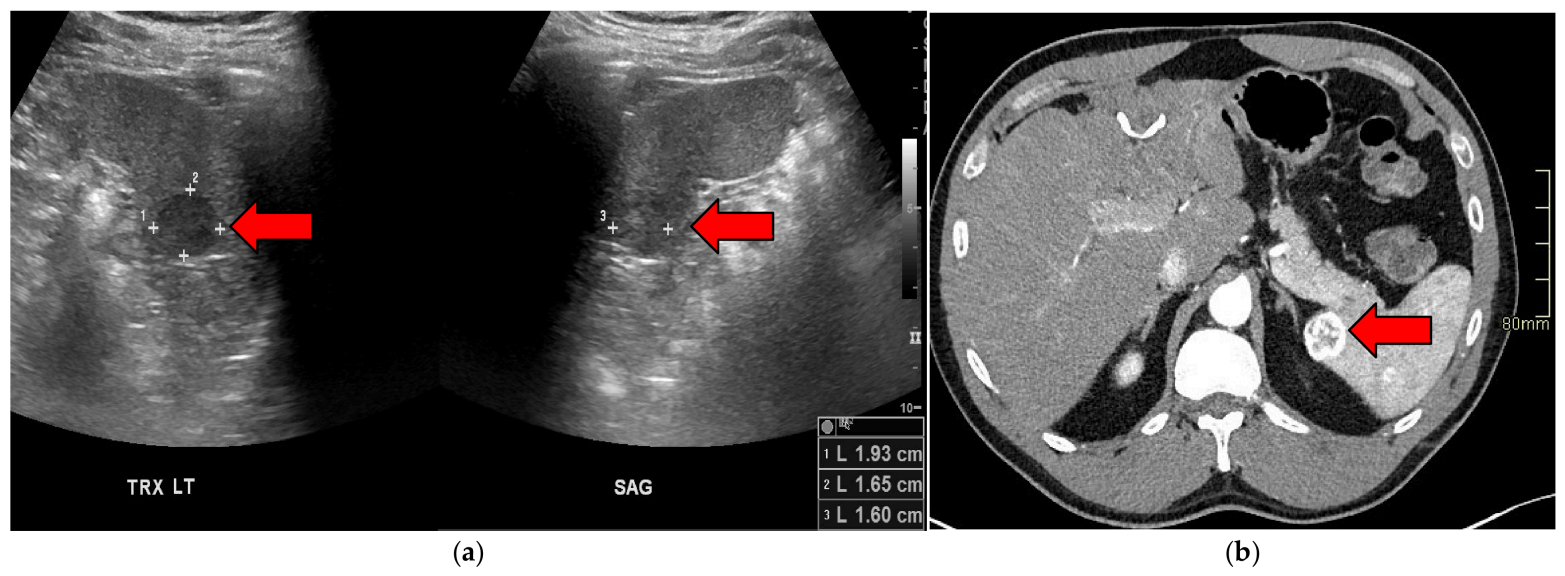
49-year-old male with *Echinococcus multilocularis*. (**a**) Ultrasound shows a well-circumscribed hypo-echoic lesion with daughter cysts. (**b**) On axial contrast-enhanced CT, there are multiple well-defined hypodense cystic lesions with peripheral and septal calcifications.

Imaging features of splenic hydatid cysts include well-defined, unilocular or multilocular cystic lesions often with internal daughter cysts, floating membranes, and occasionally rim or internal calcifications. On ultrasound, hydatid cysts typically appear as anechoic cysts with internal septations or daughter cysts (Figure 3a). CT demonstrates a sharply demarcated, low attenuation cyst, usually without enhancement, and is superior for detecting wall calcification (Figure 3b). MRI shows a homogenous T2 hyperintense, T1 hypo-intense cyst, and can demonstrate internal septations and daughter cysts, similar to other modalities [14].

### 2.4. Hematoma

Splenic hematomas most commonly occur secondary to blunt abdominal trauma and are present in 77% of patients with blunt splenic injury. The majority of splenic hematomas are seen in young adults with a strong male predominance. The most common mechanism is motor vehicle accidents, followed by falls and other blunt trauma [15]. There is no ethnic predominance with these splenic lesions [16]. On gross pathology, splenic hematomas present as broad areas of dark red or purple discoloration, with or without capsular laceration or rupture. Microscopically, the hematomas show parenchymal hemorrhage with extravasated red blood cells, fibrin deposition, and variable degrees of neutrophilic infiltrate [17].

The imaging features of splenic hematomas on contrast-enhanced CT, which is the gold standard for trauma evaluation, appear as hypodense areas relative to normal splenic parenchyma. Subscapular hematomas are seen as crescentic or lentiform collections between the splenic capsule and parenchyma. Meanwhile, intraparenchymal hematomas appear as well-defined or irregular hypodense regions within the splenic tissue [18]. On ultrasound, splenic hematomas are hypo-echoic or heterogeneous. As they age, these lesions become more echogenic and eventually anechoic as liquefaction occurs [19]. MRI can further characterize hematomas, with acute blood products appearing iso intense to hypo-intense on T1-weighted images and hyperintense on T2-weighted images [19].

### 2.5. Abscess/Micro-Abscess

The spleen is an uncommon location for abscess formation, given the relative immunoprotection by the spleen’s internal reticuloendothelial system. As a result, splenic abscesses are more common in immunosuppressed or immunocompromised individuals, most frequently via hematogenous spread of causative organisms [20,21,22]. Gram-negative bacteria, Gram-positive bacteria, fungal entities, and rarely mycobacterium including tuberculosis, have been reported, with a slightly greater incidence of Gram-negative bacteria [21,23]. The age of presentation is broad, often associated with additional underlying conditions resulting in immune compromise. While these are commonly solitary lesions, splenic abscesses may also present as multiple splenic masses, particularly in the setting of fungal microabscesses. Laboratory diagnosis is often based on blood cultures for causative organisms or can be obtained via image-guided aspiration [20,21,22]. Histopathology demonstrates neutrophilic infiltrates surrounded by immune cells and resultant fibrous deposition [24].

**Figure 4:**
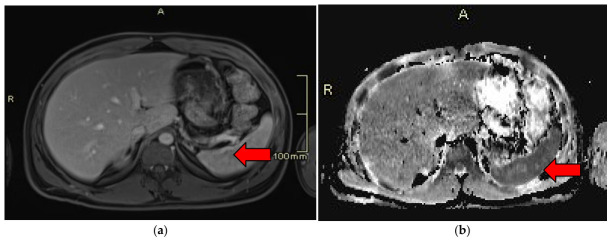
Micro-abscess in a 25-year-old male with fever and chills. (**a**) Axial T1-weighted MRI of multiple small, rounded hypo-attenuated lesions scattered throughout the spleen. (**b**) On an axial T2-weighted image, the spleen demonstrates multiple small, scattered round lesions throughout the splenic parenchyma with high fluid signal intensity, producing a diffuse “miliary” appearance.

On ultrasound, splenic abscesses typically present as masses that demonstrate central hypo-echogenicity, well-defined or ill-defined masses, and can be solitary (more commonly) or multiple [10]. The presence of gas can result in “dirty-shadowing” artifacts and may limit sonographic assessment. With CEUS, abscesses show enhancement of the wall and septa, with necrotic components showing no enhancement [10]. On CT, the masses typically present as hypoattenuating lesions and can demonstrate peripheral enhancement. On MRI, the lesion(s) typically demonstrate high T2 fluid signal centrally, with low T1 signal, although this signal may vary depending on the degree of internal proteinaceous or hemorrhagic contents (Figure 4a,b). Peripheral enhancement may develop as the collection matures [23,25].

### 2.6. Lymphangioma

Lymphangioma is a rare benign congenital tumor. It primarily affects children under two years of age, representing 80–90% of cases, with 50–65% present at birth [26]. No significant gender differences in incidence have been found. Abdominal lymphangiomas occur in less than 5% of lymphangiomas, with most being in the neck or axilla. There has been no specific ethnic predisposition documented for splenic lymphangiomas [26]. On pathology, lymphangiomas appear as thin-walled cysts varying in size, filled with clear or proteinaceous fluid. Some lesions are multilocular and lobulated. Cysts are lined by a single layer of flattened endothelial cells [27].

**Figure 5:**
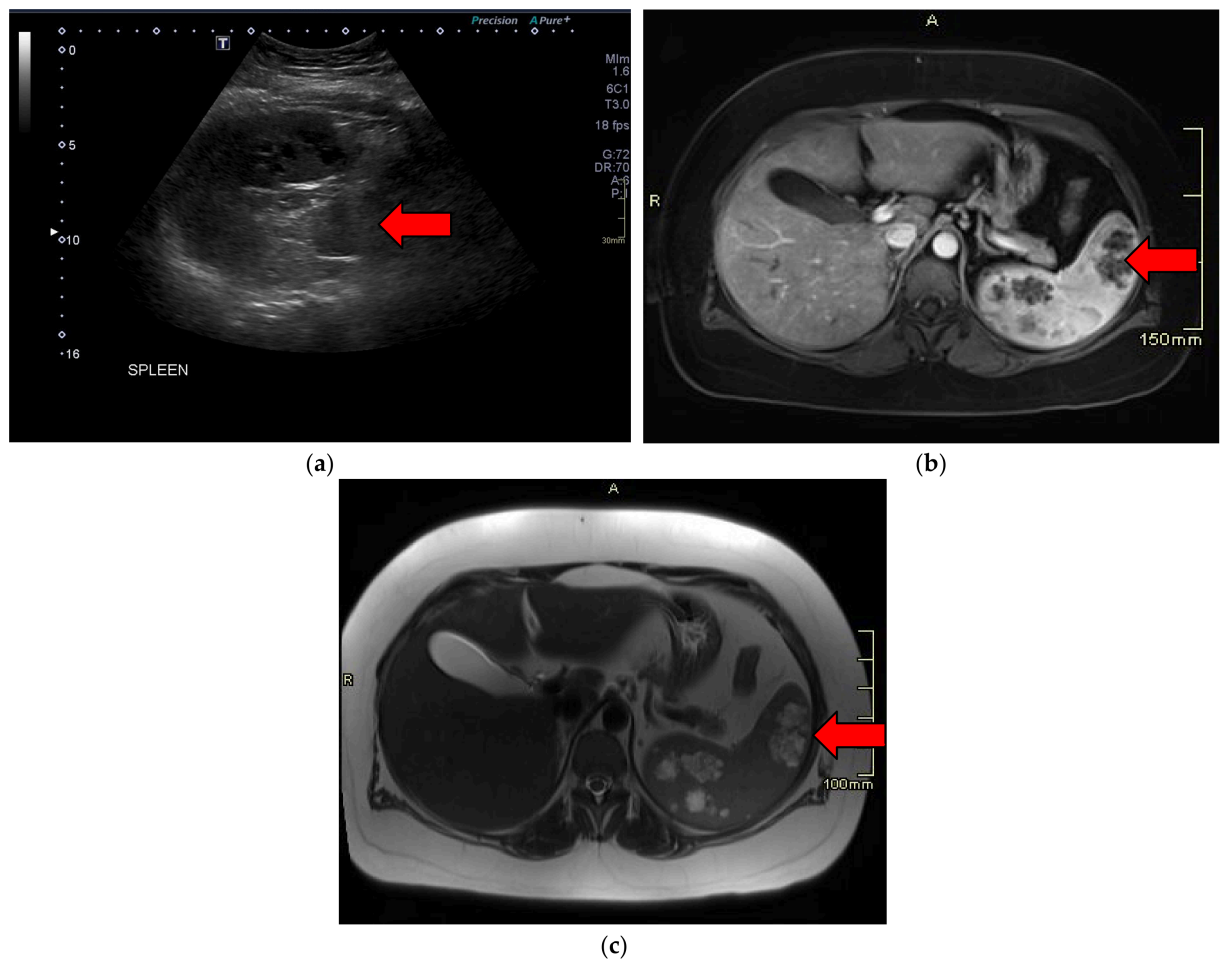
34-year-old female with incidental splenic lesion. (**a**) On long-axis ultrasound of the spleen, multiple hypo-echoic and anechoic cystic lesions with thin septations. (**b**) On an axial T1, the spleen contains well-circumscribed multiloculated cystic lesions that are hypo-intense. (**c**) On an axial T2, the lymphangioma demonstrates multiple well-defined “honeycomb” like masses with high signal intensity and are thin-walled.

Lymphangiomas are typically well-defined with thin-walled unilocular or multilocular cysts [4]. On ultrasound, lymphangiomas present as well-defined hypo-echoic or anechoic cystic lesions, with thin septations occasionally visible as seen in Figure 5a [27]. On contrast-enhanced ultrasound, the lesions demonstrate vascularization of the cyst wall with enhancement of the septa and capsule [10]. On CT, lesions are well circumscribed and have low attenuation. These lesions do not show significant enhancement following contrast administration [28]. On MRI, the lesions are multilocular and cystic with high signal intensity on T2-weighted images and typically low signal on T1-weighted images (Figure 5b,c). Prior internal hemorrhage or an intrinsic high protein content signal can result in increased T1 signal. Thin septa within the tissue appear hypo-intense [28]. Imaging can help differentiate splenic lymphangiomas from other cystic or vascular lesions, but histopathological confirmation may still be required in ambiguous cases [28].

### 2.7. Sclerosing Angiomatoid Nodular Transformation (SANT)

Sclerosing Angiomatoid Nodular Transformation (SANT) is a rare benign vascular lesion of the spleen comprising 0.007% of splenic lesions identified incidentally during surgical or autopsy examinations [29]. Most cases occur in adults between 30 and 60 years of age. There is a slight predominance of the splenic lesion in females compared to males [30]. There is no well-established ethnic distribution for SANTs [31]. Pathologically, SANTs are often altered red pulp entrapped by a stromal proliferation as a result of hemorrhage, trauma, or inflammation [29,30]. Beins, capillaries, and sinusoids can be seen on pathology, distinguishing SANTs from other splenic lesions that contain only one type of blood vessel [29]. SANTs typically appear as a single, clearly defined mass in the spleen, not surrounded by a capsule. Often these lesions demonstrate a stellate central scar [30].

**Figure 6:**
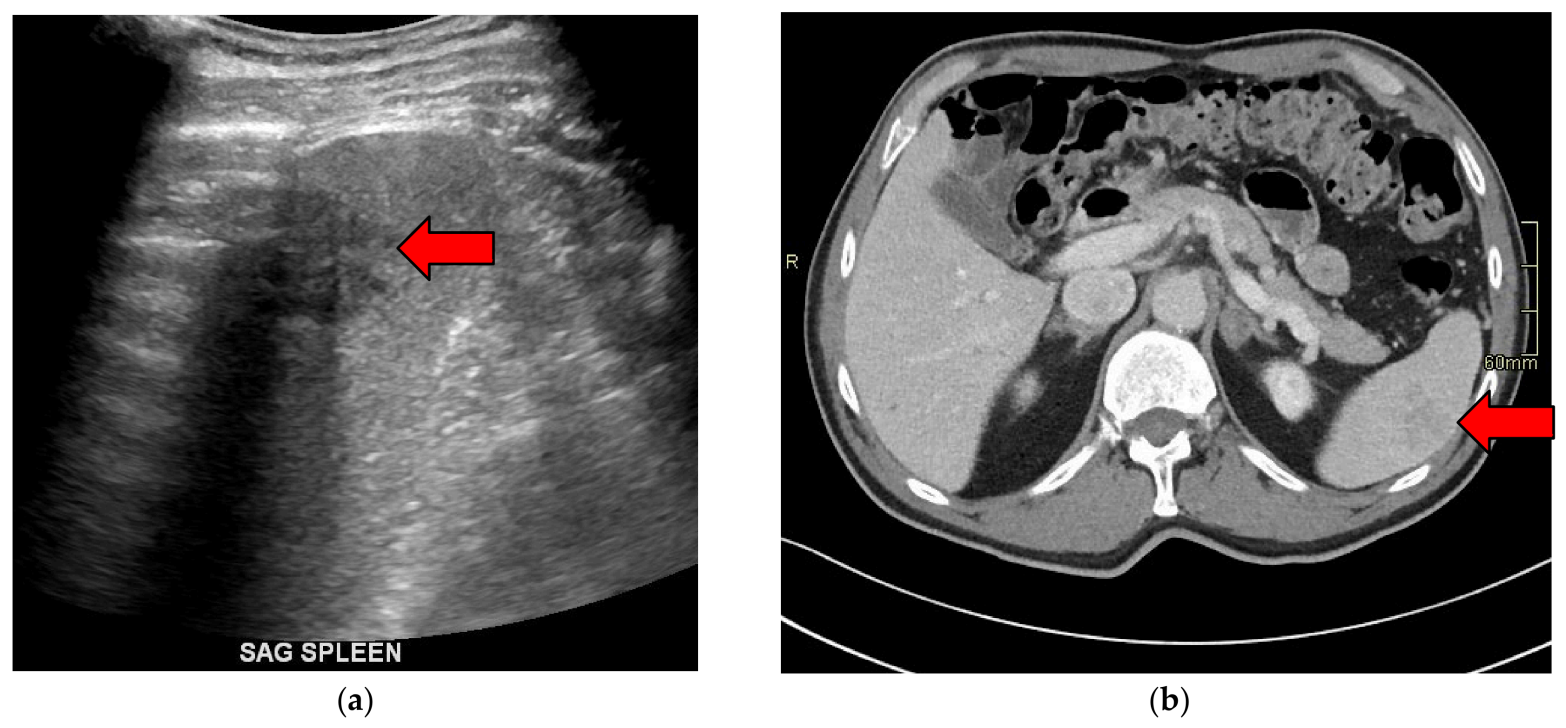

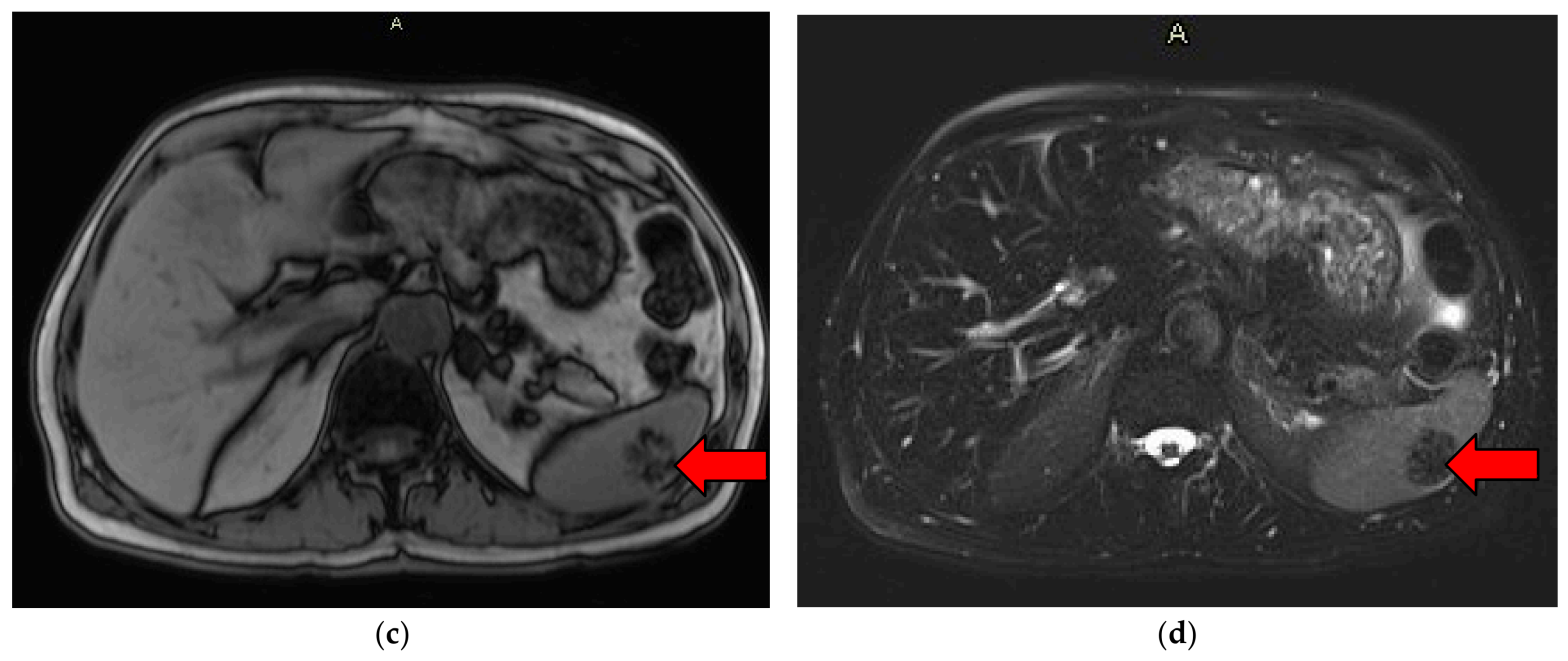
68-year-old male with an incidental splenic lesion. (**a**) Ultrasound shows a hypo-echoic splenic lesion with ill-defined margins. (**b**) On portal venous phase contrast-enhanced CT, the lesion is ill-defined, heterogeneous, and hypoattenuating. (**c**) On an axial T1 out-of-phase imaging, the lesion is well circumscribed with heterogeneous signal intensity and internal radiating septations forming a spoke wheel configuration. (**d**) On an axial T2-weighted image, the splenic lesion demonstrates low signal intensity with hypo-intense septa, forming a spoke wheel morphology.

On imaging, SANTs are solitary, well-circumscribed masses with progressive centripetal enhancement, a central fibrous scar, and a spoke wheel pattern best visualized using contrast-enhanced CT or MRI [32]. On ultrasound, the lesion can be a hypo-echoic or a heterogeneous mass with internal vascularity as seen in Figure 6a [4]. On non-contrast CT, the lesions are characterized by well-demarcated, round or lobulated mass, typically hypodense. On contrast-enhanced CT, SANT demonstrates heterogeneous hypo-enhancement in the arterial and portal venous phase, with a distinctive progressive enhancement pattern known as a “spoke wheel” pattern as seen in Figure 6b [29]. On MRI, the T2-weighted images of the lesion are hypo-intense, reflecting fibrous tissue and hemosiderin deposition, with variable T1 signal. The central scar is generally T2 hypo-intense. The spoke wheel pattern appears with peripheral and septal enhancement following gadolinium contrast administration as seen in Figure 6c,d [32].

### 2.8. Hemangioma

Hemangiomas of the spleen are the most common benign primary neoplasm of the spleen, but are overall rare in practice [33]. Up to 80% of cases are found incidentally during imaging or surgery. Hemangiomas can occur across a wide range of ages but are frequently identified in adults with a mean age of around 60 years. Splenic hemangiomas are very rare in children [33]. Most of these incidental splenic lesions are solitary and small, measuring less than 4 cm. Hemangiomas of the spleen have no ethnic or sex predominance [33]. Hemangiomas are well-circumscribed, non-encapsulated nodules composed of proliferated thin-walled vascular channels lined by endothelial cells. On macroscopic pathology, hemangiomas appear as spongy, red-blue nodules, with areas of infarction or necrosis in larger lesions [33]. The larger cavernous hemangiomas consist of large, dilated, blood-filled vascular spaces separated by thin septa [33].

**Figure 7:**
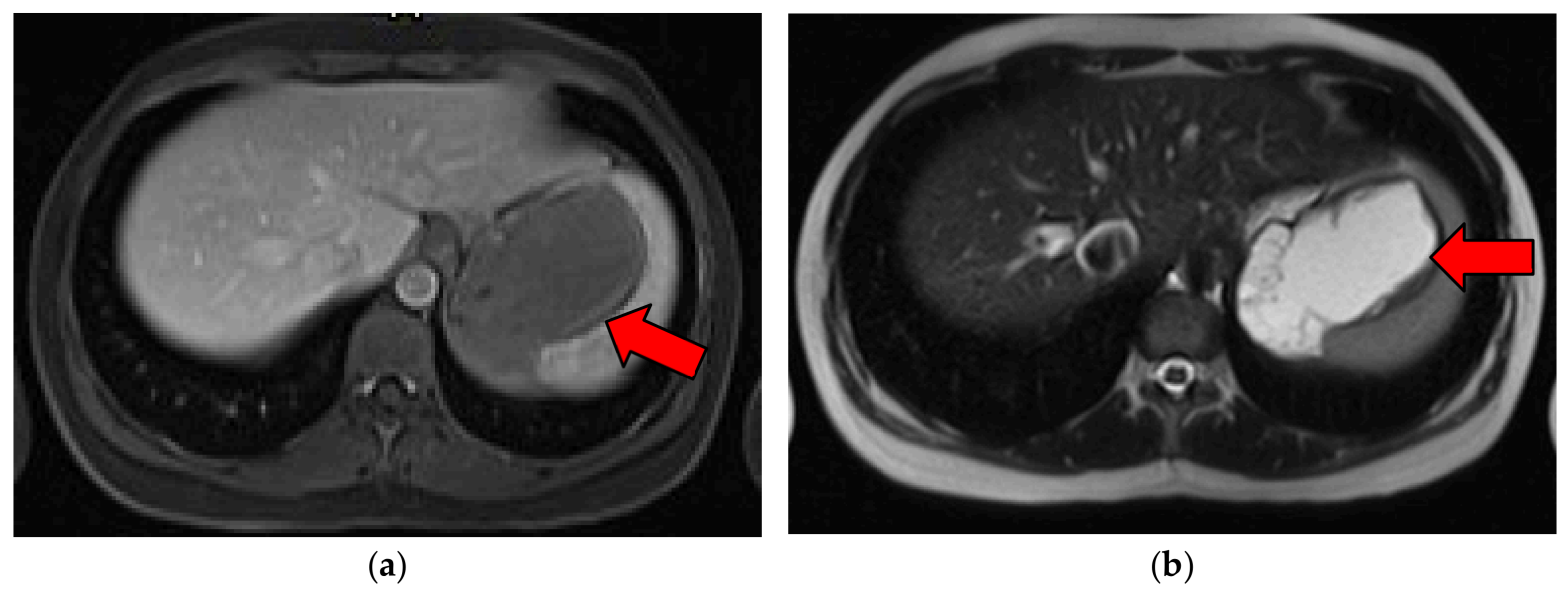
29-year-old female with an incidental enlarging lesion causing pain. (**a**) Axial post-gadolinium T1-weighted MRI demonstrates a well-defined hypo-intense lesion with subtle peripheral enhancement. (**b**) On an axial T2-weighted MRI image, the hemangioma is hyperintense with a well-circumscribed margin.

On ultrasound, splenic hemangiomas are well-defined, solitary, homogenous, and hyper-echoic [4]. Most lesions are avascular on color Doppler due to slow flow within the cavernous spaces [27]. Following contrast administration on CT or MRI, splenic hemangiomas have peripheral nodular enhancement with progressive centripetal fill-in [34]. This pattern closely mirrors hepatic hemangiomas and is similarly highly suggestive of splenic hemangiomas [4]. However, hemangiomas with fibrosis, hemorrhage, or cystic degeneration can have correspondingly variable findings on CT or MRI (Figure 7a) [34]. On T2-weighted MRI, hemangiomas classically demonstrate marked hyperintensity and are “light bulb bright”, helping distinguish them from other splenic lesions (Figure 7b). Hemangiomas have an absence of restricted diffusion on diffusion-weighted MRI, distinguishing them from other malignant lesions such as lymphoma or angiosarcoma [34].

### 2.9. Hamartoma

Splenic hamartomas are a rare benign nodular malformation with a prevalence of less than 1%. Hamartomas have no difference in prevalence with age or gender. Splenic hamartomas are generally asymptomatic [4]. Pathologically, splenic hamartomas are solitary, well-circumscribed, encapsulated lesions composed of disorganized red pulp elements. These lesions display unorganized vascular lesions of varying width. In chronic lesions, fibrosis, hemorrhage, and calcifications may be present [35].

**Figure 8:**
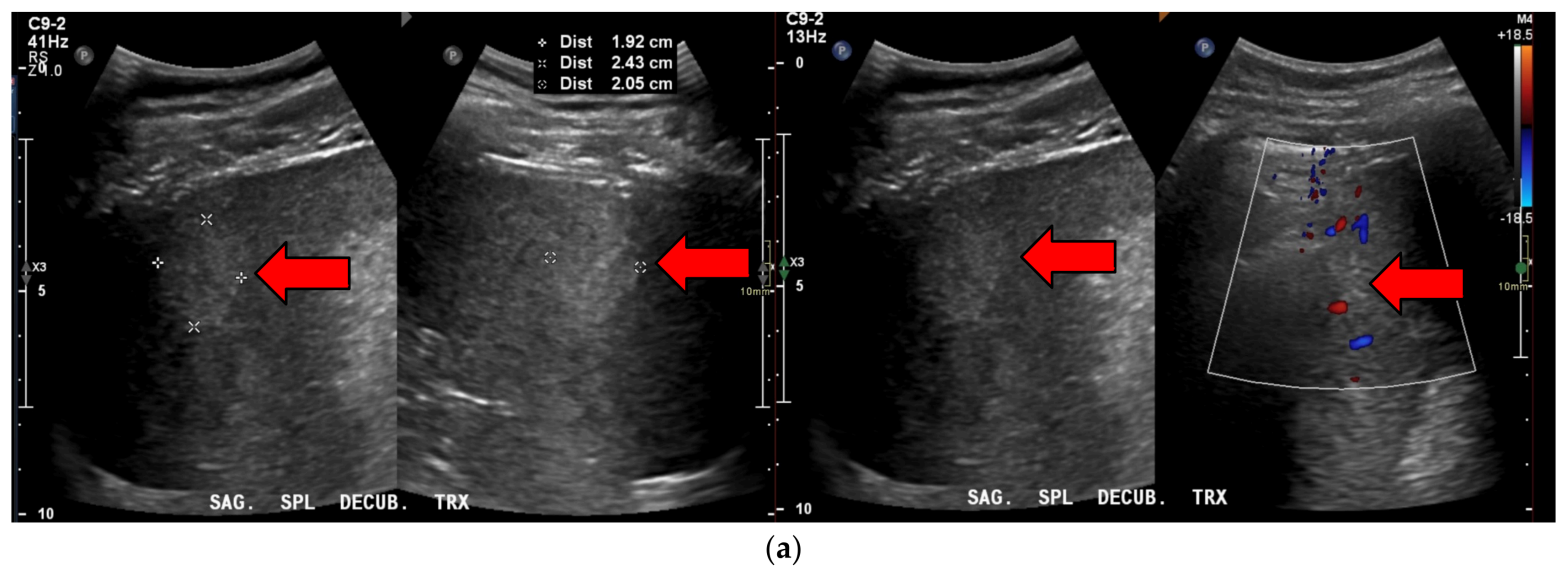

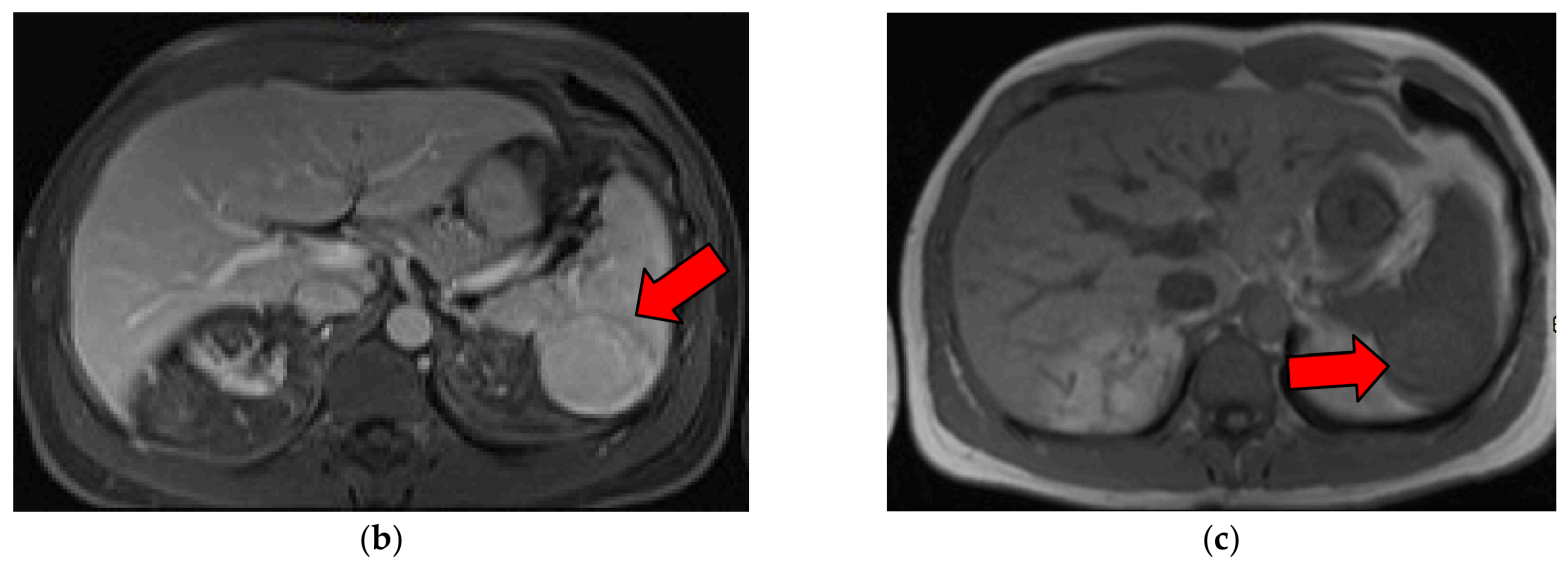
28 year old male with an incidental splenic lesion. (**a**) Ultrasound shows a mildly hyper-echoic lesion with mild vascularity. (**b**) On post-gadolinium T1 fat-suppressed imaging, there is an abnormal expansile contour of the mass with signal characteristics similar to background spleen. (**c**) On an axial pregadolinium T1 imaging, the hamartoma is well circumscribed with minimally increased T1 signal compared to the native spleen.

On imaging, splenic hamartomas are well-defined solitary solid masses. On ultrasound, splenic hamartomas appear as homogenous, hyper-echoic lesions, although they can be heterogeneous if there is internal necrosis or fibrosis (Figure 8a). Color Doppler shows increased vascularity within the lesion [34]. On non-contrast CT, the lesions are well-circumscribed, iso-to-hypodense masses without calcification. With CT contrast, these lesions show heterogeneous enhancement in the arterial phase and become progressively more homogenous and isointense to the spleen on delayed phases [36]. MRI typically shows isointensity to splenic parenchyma on T1-weighted images and mild to marked hyperintensity on T2-weighted images. Following gadolinium administration, hamartomas exhibit heterogeneous early enhancement that becomes more uniform and prolonged on delayed images (Figure 8b,c). This gradual enhancement helps distinguish splenic hamartoma from malignant lesions, which may show restricted diffusion and rapid washout [35,36].

### 2.10. Angiosarcoma

Splenic angiosarcoma is an extremely rare and highly aggressive malignancy. The median age of diagnosis is 50–65 years of age, with a slight male predominance [37]. Splenic angiosarcomas demonstrate distinctive histopathological features that include abnormal, pleomorphic malignant endothelial cells forming variable architectural patterns ranging from well-circumscribed nodules to poorly defined necrotic or hemorrhagic masses [38].

Imaging splenic angiosarcomas demonstrates variable but characteristic imaging features across multiple modalities, with the most common finding being complex masses in an enlarged spleen, accompanied by metastatic disease. On ultrasound, splenic angiosarcomas usually present as multiple hypo-echoic nodules in an enlarged spleen. On CT, the spleen shows significant enlargement with multiple nodular, ill-defined low-density lesions and central necrotic areas [39]. MRI findings are similar, with T1-weighted imaging demonstrating low-to-heterogeneous signal intensity, with foci of higher signal intensity reflecting internal areas of necrotic hemorrhage. T2-weighted imaging shows heterogeneous high signal intensity with areas of low central signal representing hemorrhage, hemosiderin deposition, or necrosis [40].

### 2.11. Lymphoma

Lymphoma of the spleen encompasses both primary lymphoma confined to the spleen and splenic hilar lymph nodes and secondary systemic splenic involvement. Primary splenic involvement is rare, accounting for less than 1–2% of all non-Hodgkin lymphomas; the spleen is frequently secondarily involved in systemic lymphomas. Among lymphomas involving the spleen, diffuse large B-cell lymphoma (DLBCL) is the most common, representing 23–46% of cases [41]. The median age for splenic lymphoma is 65 years, with a male predominance [42]. Splenic lymphomas have three distinct morphologic patterns of white pulp, red pulp, and nodular pattern [43].

**Figure 9:**
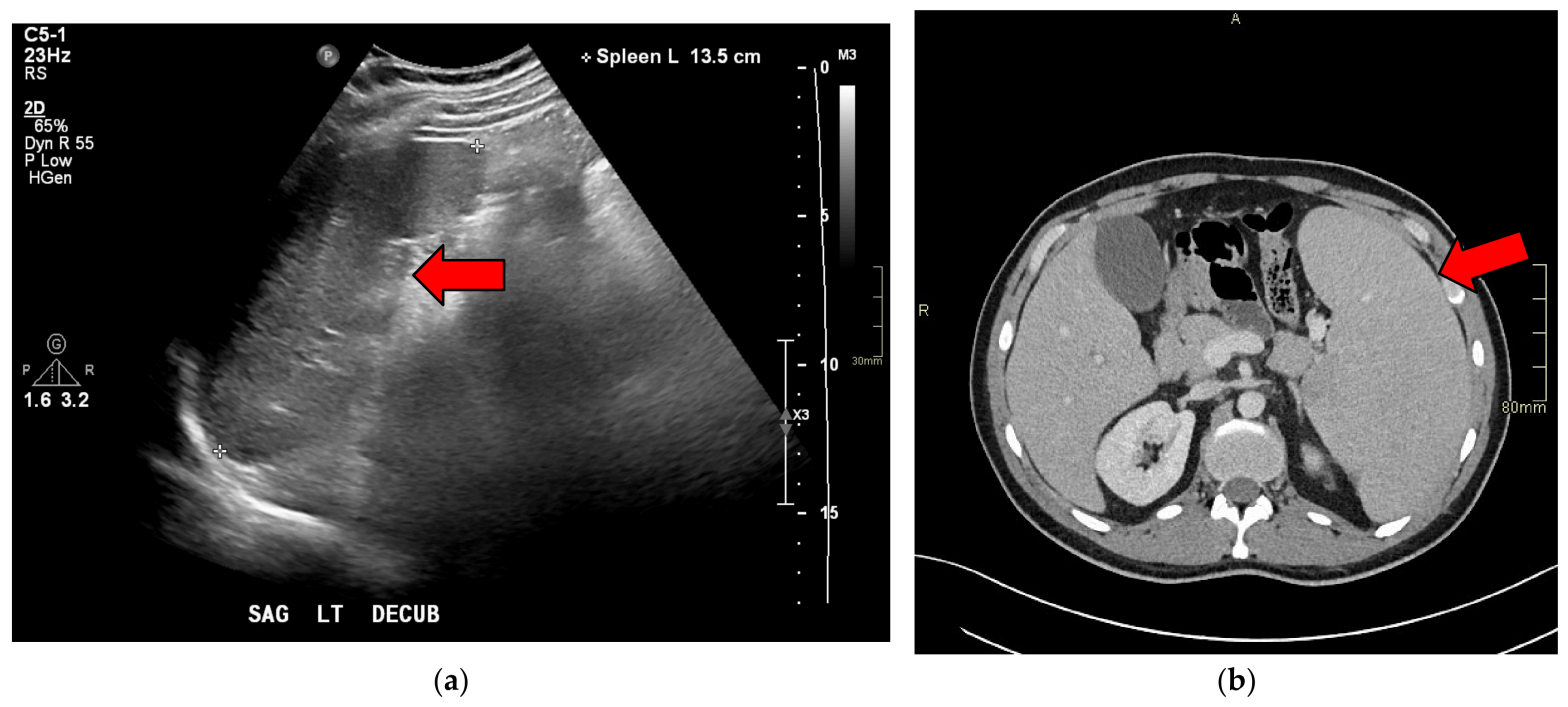
Lymphoma (**a**) On ultrasound, an enlarged spleen with hypo-echoic ill-defined lesions (**b**) On contrast-enhanced CT, the spleen is enlarged with homogenous attenuation.

Splenic lymphomas can demonstrate four imaging patterns: homogenous enlargement (type 1), miliary nodules (type 2), multifocal masses of varying size (type 3), or a solitary large mass (type 4) [44]. On ultrasound, lymphoma often is hypo-echoic and may show homogeneously echogenic enlargement or scattered multifocal masses (Figure 9a) [4]. On CT, splenic lymphoma appears hypodense relative to splenic parenchyma with mild enhancement in most cases as seen in Figure 9b [44]. On MRI, splenic lymphomas show areas of low signal intensity on both T1-weighted and T2-weighted images, though T2-weighted images may show higher signal intensity in lesions with dense fibrosis [45]. On diffusion-weighted imaging, lymphoma demonstrates increased diffusion restriction compared to adjacent splenic parenchyma. If present, multifocal splenic lesions may show focal diffusion restriction or altered enhancement compared to the background splenic parenchyma [4].

### 2.12. Inflammatory Pseudotumor

A reactive tumor-like lesion that occurs throughout the body, an inflammatory pseudotumor can rarely be seen in the spleen. Inflammatory pseudotumors show a female predominance and are more common in middle-aged or older adults [46]. Splenic inflammatory pseudotumors are associated with Epstein–Barr virus (EBV), with corresponding geographic predominance in East Asia due to background prevalence of EBV [46,47]. On pathology, splenic inflammatory pseudotumors are composed of inflammatory cells and/or spindle cell proliferation with interspersed collagenous stroma. The presence of spindle cells is suggestive of EBV-positive lesions [46,47].

**Figure 10:**
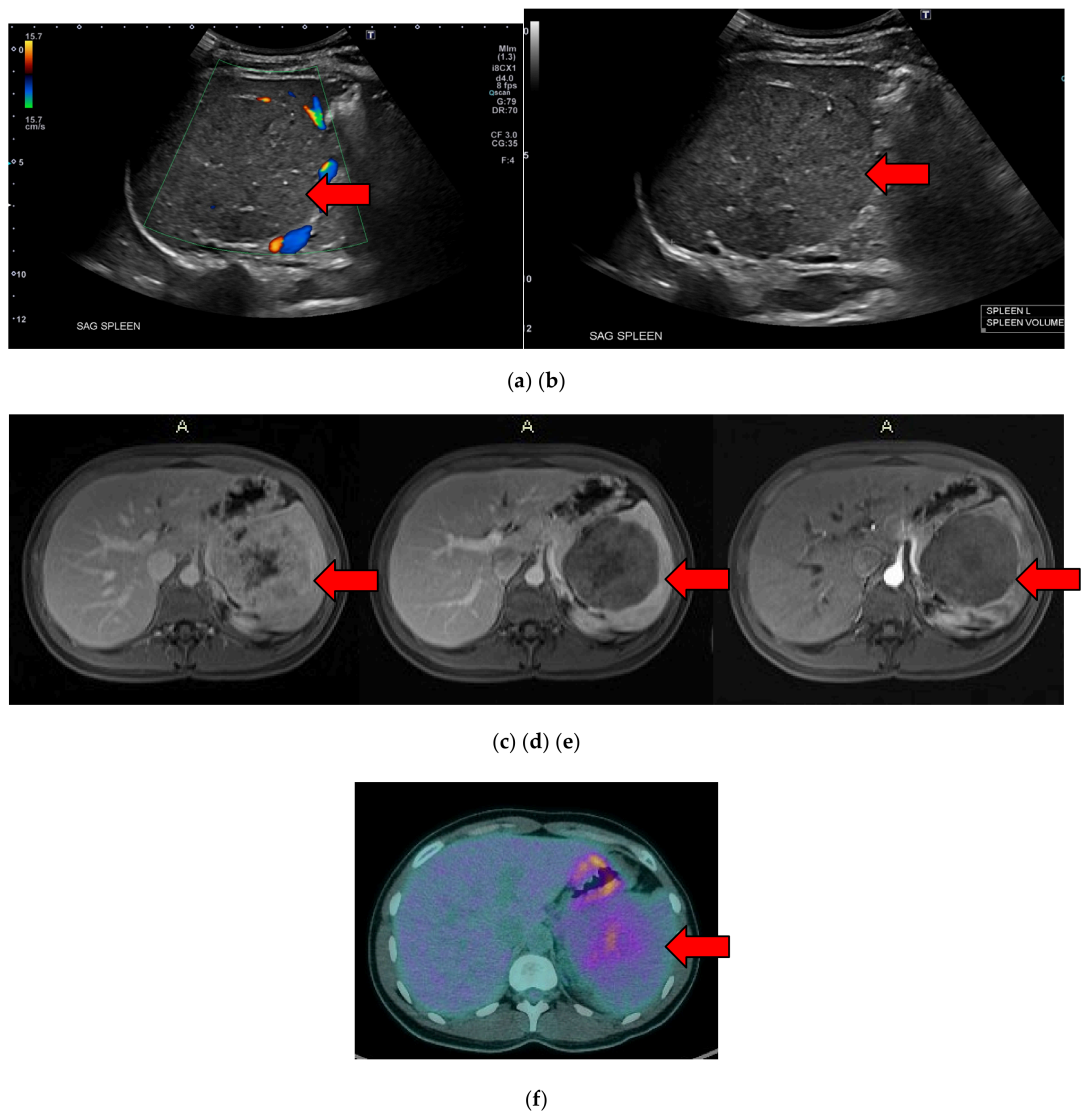
Post-traumatic pseudotumor. (**a**,**b**) Ultrasound demonstrates a well-circumscribed splenic mass with some peripheral vascularity upon Doppler interrogation. (**c**–**e**) MRI demonstrates gradual enhancement of the central fibrous components on post-gadolinium T1 fat-suppressed MRI. (**f**) PET/CT demonstrates mild FDG avidity, greater than the background liver.

Splenic inflammatory pseudotumors have variable imaging features that overlap with malignant splenic tumors; histopathological confirmation is often required for a definitive diagnosis. On ultrasound, lesions often appear as well-circumscribed hypo-echoic solid-like masses. If lesions are ill-defined, multiple, necrosed, or fibrosed, they can appear diffusely heterogeneous as seen in Figure 10a,b [4]. On CT, these lesions can be difficult to differentiate from splenic parenchyma without contrast. However, with CT contrast, the splenic lesions are large well-circumscribed hypo-enhancing solid-appearing masses [4]. On MRI, splenic inflammatory pseudotumors show low-to-moderate signal intensity on T1-weighted images and heterogeneous signal intensity on T2-weighted images, depending on fibrous and necrosis tissue as seen in Figure 10c–e [47]. Delayed enhancement may be seen on post-gadolinium MRI due to the often abundant background fibrous tissue (Figure 10f) [4].

### 2.13. Metastases

Splenic metastases are uncommon, with an incidence of 2.3–7.1% in cancer patients, representing less than 1% of all metastases [48]. The spleen is considered a relatively protected organ from metastatic disease due to anatomic and immunologic factors [49]. The most common primary tumors to metastasize to the spleen include tumors of breast, lung, ovarian, melanoma, and colorectal origin [4]. On pathology, splenic metastases demonstrate variable pathologic features of either solitary, multiple, or diffuse lesions depending on the primary tumor [48]. Immunohistochemistry is used to phenotype metastatic tumors that are poorly differentiated [4].

**Figure 11:**
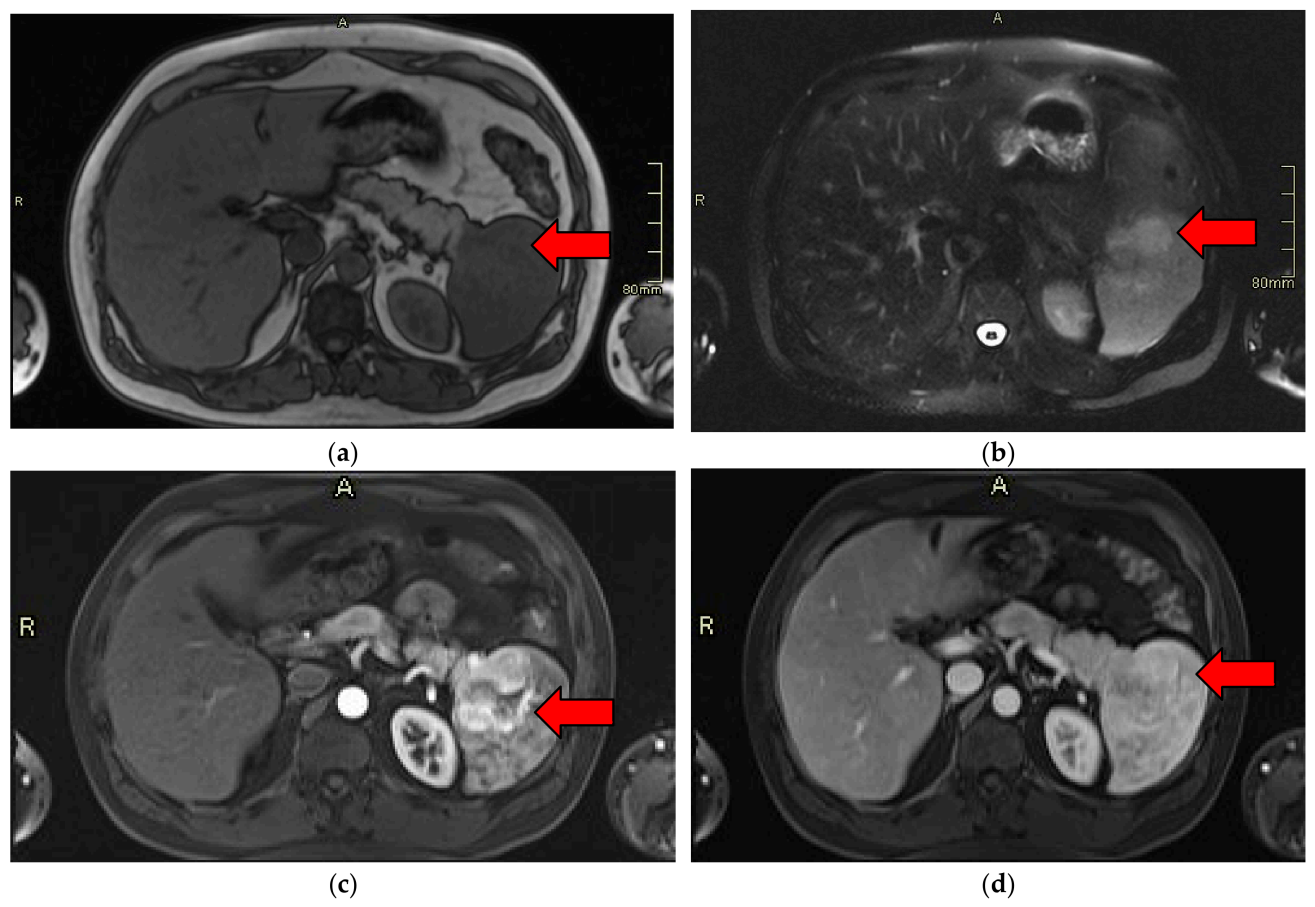
A 43-year-old man presented with abdominal pain with an incidental splenic lesion. (**a**) T1 imaging demonstrates an isointense splenic lesion. (**b**) T2 fat-suppressed images demonstrate mild T2 hyperintensity of the lesion. (**c**) The lesion demonstrates heterogeneous avid early arterial enhancement. (**d**) On late arterial phase, the lesion becomes more homogenous to background spleen.

On ultrasound, splenic metastases are predominantly hypo-echoic, though they can have mixed echogenicity or be hyper-echoic. Contrast-enhanced ultrasound shows variable enhancement [10]. With CT, these lesions may be hypoattenuating cystic lesions or can be more solid with variable enhancement. On MRI, the lesions are often T2 hyperintense and T1 hypo-intense with variable enhancement (Figure 11a–d) [4]. In one study, restricted diffusion was present in 50% of malignant lesions and 0% of benign lesions, suggesting DWI may be highly specific [48]. Ultimately, the features of the splenic metastasis are highly variable regardless of imaging modality, often mirroring the primary tumor.

### 2.14. Littoral Cell Angioma

Splenic littoral cell angioma (LCA) is an exceedingly rare primary vascular neoplasm of the spleen. LCA demonstrates a slight female predominance and usually occurs in adults with a mean age at diagnosis of 56 years [50]. The tumor is more commonly characterized by multiple lesions rather than solitary lesions, with 50% of patients asymptomatic at diagnosis. No geographic, ethnic, or familial predilection has been established in the literature [50]. The key pathological features of splenic littoral cell angiomas are anastomosing, sinus-like vascular channels lined by tall endothelial cells with conspicuous cytoplasmic hemosiderin and evidence of hemophagocytosis, lacking cellular atypia and with very low mitotic activity [51].

Splenic LCA demonstrates non-specific imaging features, typically presenting as multiple hypoattenuating masses in an enlarged spleen on CT [52,53]. LCA on ultrasound appears as ill-defined echogenic lesions without acoustic enhancement [54]. On non-contrast CT, lesions appear isoattenuating or slightly hypoattenuating relative to normal splenic parenchyma [53]. Contrast-enhanced CT demonstrated hypoattenuating masses relative to the normal spleen in the arterial phase with enhancement [54]. There is a progressive “filling lake” enhancement during venous and delayed phases, with some lesions showing peripheral ring enhancement. In multiple lesions, there is a “less–more–less” pattern where the number of visible lesions varies across phases [53]. On MRI T1-weighted images, there is equal to low signal intensity with mixed signals. On MRI, T2-weighted imaging, there are high and low mixed signals with a characteristic “freckle sign” reflecting hemosiderin deposition within the lesions [53].

## 3. Conclusions

**Figure 12:**
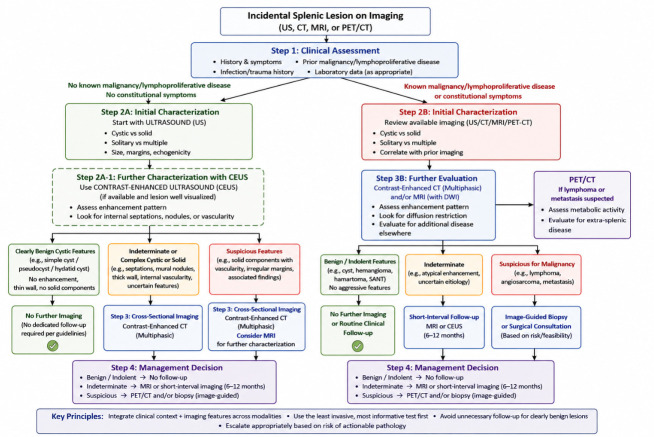
Algorithm for diagnosing incidental splenic lesions.

In conclusion, the overlapping imaging features of many incidental splenic lesions can make definitive diagnosis challenging. As described, key imaging features and pertinent patient demographics/clinical features may play an important role in accurate diagnosis (Table A1). Identification of clearly benign features, or conversely worrisome features, aids in subsequent clinical decision-making, as well as downstream investigation or intervention. Although imaging features can be highly suggestive of a given diagnosis, atypical lesions often require image-guided biopsy or surgical excision for definitive histopathologic diagnosis (Figure 12) [55]. For such non-benign lesions, contrast-enhanced ultrasound, contrast-enhanced CT, and contrast-enhanced MRI demonstrate high diagnostic accuracy [56].

## Data Availability

The original contributions presented in this study are included in the article. Further inquiries can be directed to the corresponding authors.

## Abbreviations

The following abbreviations are used in this manuscript:

CT	Computed Tomography
EBV	Epstein–Barr Virus
LCA	Littoral Cell Angioma
MRI	Magnetic Resonance Imaging
PET	Positron Emission Tomography
SANT	Sclerosing Angiomatoid Nodular Transformation
US	Ultrasound
CEUS	Contrast-Enhanced Ultrasound

## Appendix A

**Table A1:** Summary of splenic lesions epidemiology, clinical presentation, and imaging features.

Lesion	Epidemiology/Clinical Features	Imaging Features
Solitary Cystic Lesions		
Primary (epithelial) cyst	10% of benign nonparasitic splenic cysts Female predilection More common in children and young adults Asymptomatic	Ultrasound: well-defined, unilocular or multilocular, thin-walled anechoic lesions on ultrasound. No internal solid components or vascularity. CT: homogenous low attenuation well-circumscribed thin-walled masses. MRI: Homogeneous, high signal intensity on T2-weighted images. Low signal intensity on T1-weighted images.
Pseudocyst	70–80% of benign nonparasitic splenic cysts Female predilection Asymptomatic	Ultrasound: Well defined and hypo-echoic CT: Well-circumscribed, unilocular, and sometimes multilocular with fluid attenuation. MRI: Variable T1 signal intensity depending on the contents, being proteinaceous or hemorrhagic. T2 hyperintense
Hydatid Cyst	0.5–0.8% of echinococcal presentations Common outside North America More common in adults, and no sex predilection Asymptomatic	Ultrasound: Anechoic cysts with internal septation or daughter cysts CT: Well-circumscribed hypo-attenuated cyst. Superior for detecting wall calcification MRI: Homogeneous on MRI. Hyperintense on T2-weighted imaging and hypo-intense on T1-weighted imaging.
Hematoma	Commonly occur as a result of blunt abdominal trauma. Majority in young adults with male predominance	Ultrasound: hypo-echoic or heterogeneous CT: Hypoattenuating, non-enhancing MRI: Acute blood products appear isotense to hypodense on T1-weighted images, while hyperintense on T2-weighted images.
Multiple Cystic Lesions		
Abscess/micro-abscess	More common in immunosuppressed or immunocompromised individuals Broad age range Commonly present with abdominal pain, fever, nausea, vomiting, or chills	Ultrasound: Central hypoechogenicity, ill-defined margins CT: Hyperattenuating, often with peripheral enhancement, MRI: T2 hyperintense, T1-weighted images often have low signal intensity that can vary depending on the contents.
Lymphangioma	Benign congenital tumor 80–90% of cases in children under two years old	Ultrasound: Well-defined hypo-echoic or anechoic cystic lesions. CT: Well circumscribed and hypo-attenuated. MRI: Multilocular and cystic. On T2-weighted imaging has high signal intensity, and on T1-weighted imaging low signal intensity.
Solitary Solid Vascular Lesions		
Hamartoma	No significant gender or age differences Asymptomatic	Ultrasound: Homogenous, hyper-echoic lesions. CT: On non-contrast CT are well-circumscribed iso-to-hypodense masses. On contrast-enhanced CT, heterogenous enhancement in the arterial phase and more homogenous on delayed phase. MRI: On T1-weighted images, lesions are isodense. On T2-weighted images mild to marked hyperintensity. With gadolinium, demonstration has early heterogeneous enhancement.
Hemangioma	Commonly around the age of 60 years old. Rare in children No ethnic or gender predominance	Ultrasound: Well-defined, solitary, homogenous, and hyper-echoic masses. CT: Have peripheral nodular enhancement with progressive centripetal fill-in. MRI: Lesions have marked hypersensitivity on T2-weighted imaging. Limited restricted diffusion on DWI.
SANT	Common in adults aged 30 to 60 years old Slight female predominance No ethnic predisposition	Ultrasound: hypo-echoic or heterogeneous mass with internal vascularity CT: Non-contrast CT lesions are well-demarcated, round, or lobulated masses that are hypodense. Contrast-enhanced CT shows heterogeneous hypo-enhancement in the arterial and venous phase. Progressive “spoke wheel” enhancement. MRI: On T2-weighted imaging, the lesions are hypo-intense. Have a variable T1-weighted signal. “Spoke wheel enhancement” post-gadolinium
Angiosarcoma	Extremely rare and highly aggressive malignancy. Median age of 50–65 years old, with male predominance.	Ultrasound: multiple hypo-echoic nodules in an enlarged spleen. CT: multiple ill-defined nodules, variable density, central necrotic areas. MRI: T1-weighted imaging shows low and heterogeneous signal intensity. T2-weighted imaging shows heterogeneous high signal intensity.
Solitary Solid Nonvascular Lesions		
Lymphoma	DLBCL is the most common splenic lymphoma, representing 23–46% of cases Median age 65 years old	Ultrasound: broad range of findings, including multiple hypo-echoic splenic masses, homogenous splenic enlargement CT: Lesions appear hypodense with mild enhancement MRI: low signal intensity on both T1-weighted and T2-weighted images. T2-weighted images may show higher signal intensity in lesions with dense fibrosis.
Inflammatory pseudotumor	Female predominance is more common in middle-aged or older adults. Associated with EBV	Ultrasound: Well-circumscribed hypo-echoic solid-like masses CT: large well-circumscribed hypo-enhancing solid masses. MRI: On T1-weighted images, low-to-moderate signal intensity, and heterogeneous signal intensity on T2-weighted images.
Multiple Solid Lesions		
Littoral cell Angioma	Rare primary vascular neoplasm of the spleen. Mean age 56 years. Slight female predominance.	Ultrasound: ill-defined echogenic lesions with acoustic enhancement CT: Lesions appear iso attenuating or slightly hypoattenuating. MRI: T1 shows equal to low signal intensity with mixed signals. T2 signal variable depending on internal contents.
Metastases	Represents 1% of all metastases	Ultrasound: Predominantly hypo-echoic CT: Lesions are hypoattenuating cysts or can be more solid with variable enhancement MRI: T1-weighted imaging is hypo-intense, while T2-weighted imaging is hyperintense
